# Preconditioning strategies for improving the survival rate and paracrine ability of mesenchymal stem cells in acute kidney injury

**DOI:** 10.1111/jcmm.14035

**Published:** 2018-11-28

**Authors:** Lingfei Zhao, Chenxia Hu, Ping Zhang, Hua Jiang, Jianghua Chen

**Affiliations:** ^1^ Key Laboratory of Kidney Disease Prevention and Control Technology, Kidney Disease Center, The First Affiliated Hospital, College of Medicine Zhejiang University Hangzhou Zhejiang PR China; ^2^ Institute of Nephrology Zhejiang University Hangzhou Zhejiang PR China; ^3^ State Key Laboratory for Diagnosis and Treatment of Infectious Diseases, The First Affiliated Hospital, College of Medicine Zhejiang University Hangzhou Zhejiang PR China

**Keywords:** acute kidney injury, mesenchymal stem cells, preconditioning strategy, survival and paracrine ability

## Abstract

Acute kidney injury (AKI) is a common, severe emergency case in clinics, with high incidence, significant mortality and increased costs. Despite development in the understanding of its pathophysiology, the therapeutic choices are still confined to dialysis and renal transplantation. Considering their antiapoptotic, immunomodulatory, antioxidative and pro‐angiogenic effects, mesenchymal stem cells (MSCs) may be a promising candidate for AKI management. Based on these findings, some clinical trials have been performed, but the results are contradictory (NCT00733876, NCT01602328). The low engraftment, poor survival rate, impaired paracrine ability and delayed administration of MSCs are the four main reasons for the limited clinical efficacy. Investigators have developed a series of preconditioning strategies to improve MSC survival rates and paracrine ability. In this review, by summarizing these encouraging studies, we intend to provide a comprehensive understanding of various preconditioning strategies on AKI therapy and improve the prognosis of AKI patients by regenerative medicine.

## 1 INTRODUCTION

Acute kidney injury (AKI), defined as an abrupt decline in glomerular filtration, remains a worldwide public health issue due to its high incidence and significant mortality. It has been reported that the morbidity rate is approximately 5%‐7% in hospitalized patients and over 30% in ICU hospitalized patients.^1^, ^2^ The mortality rate of patients with AKI is approximately 50%; in cases that require dialysis therapy, the mortality rate could reach 88%.^3^ A variety of causes may induce AKI, including renal ischemia, drug nephrotoxins and sepsis. The complex pathophysiologic mechanism of AKI is also not very clear. All of these issues pose a challenge to physicians for AKI treatment.

Currently, therapeutic choices are still confined to dialysis and renal transplantation, which are limited due to high expense and shortage of donor organs.^4^ Breakthroughs in stem cell‐based therapy over the last decades may bring hope to the millions of people who suffer from this disease around the world. While pharmacologic interventions often target only a single aspect of the highly complex pathophysiology following AKI, stem cell‐based therapies may have the advantage of acting through multiple mechanisms to promote tubular epithelial cell repair.^5^ Among a variety of stem cells, mesenchymal stem cells (MSCs) have emerged as the most promising candidates for AKI therapy given their low immunogenicity, high multipotential differentiation ability, invasiveness of isolation and abundant distribution.^6^, ^7^, ^8^

Despite the encouraging results of MSCs usage in animal models, a huge gap exists between scientific observation and clinical application. In 2017, Swaminathan et  al provided a phase 2, randomized, double‐blind, multicenter trial on the use of MSCs in patients with post‐cardiac surgical AKI (NCT01602328).^9^ After randomizing 156 adult subjects, they found that time to renal function recovery, need for dialysis, and 30‐day all‐cause mortality were all compatible between the two groups.

**Figure 1:**
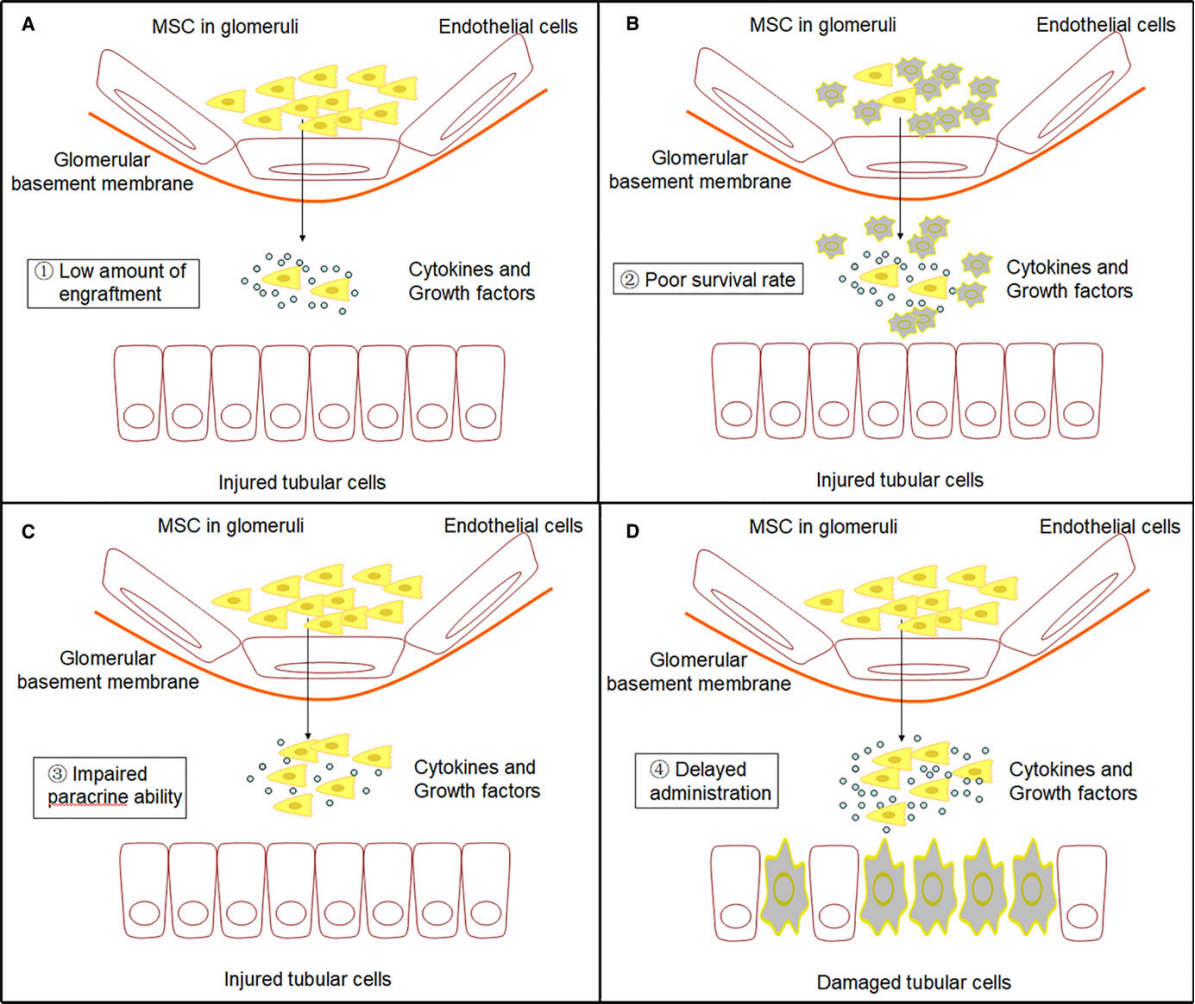
The four main factors that limit the clinical efficacy of MSCs‐based therapy. (A) The low amount of engraftment. Most delivered MSCs are trapped in unwanted organs, such as liver, lungs, and spleen. Only 1% of transplanted cells can engraft into the target tissues. (B) Poor survival rate. It is reported that more than 80%‐90% of grafted cells will die within the first week after injection due to the harsh environment in vivo. (C) Impaired paracrine ability. The regenerative effect of MSCs largely relies on their paracrine action. Impaired MSC potency/biological activity in vivo has also been reported. (D) Delayed administration. Diagnosis of AKI is still on the basis of a rise in creatinine, which may cause a delayed administration of MSCs and induce the injured kidneys to the “point of no return”

What makes MSCs lose their magic power clinically? There is growing evidence that the regenerative effect of MSCs might be mediated predominantly by paracrine action, rather than direct differentiation into target cells.^10^, ^11^, ^12^, ^13^ Once injected into an injured tissue, MSCs face a harsh environment, including reactive oxygen species (ROS) and anoikis, which are largely generated after AKI that may promote MSC apoptosis.^14^, ^15^, ^16^ It is reported that more than 80%‐90% of grafted cells will die within the first week after injection,^17^ and the remaining 9%‐19% cells may be trapped in liver, lungs and spleen.^18^ Impaired MSC potency/biological activity in vivo was also reported. Silva et  al concluded in their article that the limited clinical efficacy of MSCs might result from the low amount of engraftment, poor survival rate, impaired paracrine ability and delayed administration^19^ (Figure 1).

**Figure 2:**
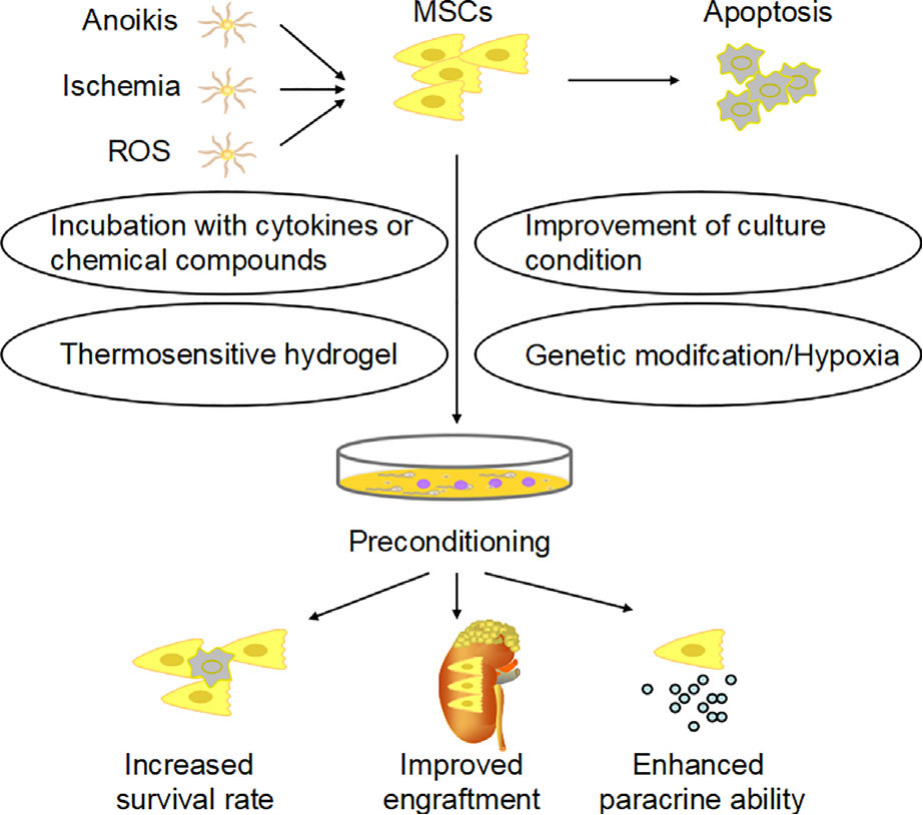
Once injected into an injured tissue, MSCs face a harsh environment, including ROS, ischemia and anoikis, which may further induce cell apoptosis. Various preconditioning strategies, such as incubation with cytokines or chemical compounds, improvement of culture condition, thermosensitive hydrogel and genetic modification, can improve the survival rate and paracrine ability of MSCs and help them migrate to the target tissue in vivo

To overcome this obstacle, some approaches to improve the ability of grafted MSCs have been explored in recent years. Investigators try to increase the number of injected cells, but it may be risky due to disturbance in blood flow causing embolism problems.^20^ Others attempt to inject cells directly into the damaged tissue, but the invasive procedures include a high risk of haemorrhage, and the number of injected MSCs is also inaccurate, as most of the cells may escape from the injected site.^21^, ^22^ Preconditioning is a promising strategy for optimizing MSCs before their transplantation. Based on the way MSCs operate, these strategies are designed to increase the survival rate of MSCs in injured tissues, enhance their paracrine ability or help them migrate to the target tissue (Figure 2). Previously, we have discussed those preconditioning strategies for enhancing the migratory ability of MSCs in AKI.^23^ In this review, we focus on summarizing the different preconditioning strategies for increasing the MSCs survival rate or paracrine ability in AKI models. Only articles that demonstrated a clear mechanism are included in our review. We look forward a bright future in which the preconditioning strategy can be used to increase the function of MSCs and, consequently, to achieve long‐term benefits of MSCs therapies in AKI patients.

## 2 STRATEGIES TO IMPROVE MSCS SURVIVAL RATES

The low survival rate of transplanted MSCs remains an important limitation for MSC therapy.^17^, ^24^ Anoikis, ischemia, inflammation and imbalance between ROS and antioxidant are likely the major causes of cell death following transplantation.^25^, ^26^, ^27^ Some preconditioning strategies have been proven to protect MSCs from harmful environments. These strategies include incubation with cytokines or chemical compounds, improvement of culture condition, thermosensitive hydrogel and genetic modification (Table 1).

**Table 1:** Strategies to improve MSCs’ survival rate in AKI

References	Year	Animal	AKI model	MSCs source	Preconditioning strategy	Outcomes
Tian et al^29^	2012	Mice	I/R	NM	Incubation with cytokines or chemical compounds	↑Survival rate; ↓Apoptosis
Masoud et al^30^	2012	Rats	I/R	BMSCs	Incubation with cytokines or chemical compounds	↑Survival rate; ↓Apoptosis
Cai et al^31^	2014	Rats	I/R	BMSCs	Incubation with cytokines or chemical compounds	↑Survival rate
Mias et al^27^	2008	Rats	I/R	BMSCs	Incubation with cytokines or chemical compounds	↑Survival rate and antioxidant; ↓Apoptosis
Liu et al^32^	2014	Rats	Gentamicin	BMSCs	Incubation with cytokines or chemical compounds	↑Proliferative index
Xinaris et al^35^	2013	Mice	Cisplatin	BMSCs	Incubation with cytokines or chemical compounds	↑Survival rate
Xu et al^39^	2016	Rats	I/R	AMSCs	Improvement of culture condition	↑Survival rate, ECM, ROS‐scavenging proteins, Bcl‐2 and pro‐survival protein phosphorylated AKT
Gao et al^44^	2012	Rats	I/R	AMSCs	Thermosensitive hydrogel	↑Survival rate
Feng et al^45^	2016	Mice	I/R	AMSCs	Thermosensitive hydrogel	↑Survival rate; ↓Apoptosis
Liu et al^51^	2015	Rats	I/R	BMSCs	Genetic modification	↑Survival rate, anti‐apoptosis, antioxidant and anti‐inflammatory
Liu et al^52^	2018	Rats	I/R	BMSCs	Genetic modification	↑Cell proliferation, activation of PI3K/Akt and MEK/ERK pathways
Mohammadzadeh‐Vardin et al^55^	2015	Rats	Cisplatin	BMSCs	Genetic modification	↑Cell viability
Hagiwara et al^58^	2008	Rats	I/R	BMSCs	Genetic modification	↓Apoptosis

AKI: acute kidney injury; I/R: ischemia/reperfusion; NM: not mentioned; BMSCs: bone marrow‐derived mesenchymal stem cells; AMSCs: adipose‐derived mesenchymal stem cells; ECM: extracellular matrix; ROS: reactive oxygen species; Bcl‐2: B cell lymphoma 2.

### 2.1 Incubation with cytokines or chemical compounds

Various cytokines or chemical compounds have been proven to have cell protective effects, and part of the mechanism is through the PI3K/AKT signalling pathway. AKT activation can promote cell survival, proliferation, growth and changes in cellular metabolic pathways through its numerous downstream targets.^28^ Tian et  al identified a new docosahexaenoic acid‐derived (DHA‐derived) lipid mediator, 14S,21Rdihydroxy‐docosa4Z,7Z,10Z,12E,16Z,19Z‐hexaenoic acid (14S,21R‐diHDHA). After incubation, MSCs showed more resistance to apoptosis in vivo and in vitro and presented more amelioration of renal ischemia/reperfusion (I/R) injury in a mouse model. The authors also demonstrated that the mechanism promoting the viability of MSCs was the activation of the PI3K/AKT signalling pathway.^29^ Another study involving the cell protective role of the PI3K/AKT signalling pathway was published by Masoud et  al in 2012. They reported that preconditioning of MSCs with S‐nitroso N‐acetyl penicillamine (SNAP), a NO donor, enhanced their proliferation, survival and engraftment in ischemic kidney, accompanied by many fold increase in the expression of AKT and B cell lymphoma 2 (Bcl‐2).^30^

Investigators also used drugs or health care products to incubate MSCs, with excellent results. Cai et  al pretreated MSCs with atorvastatin. They found that through suppression of TLR4 signalling, atorvastatin significantly increased the viability of implanted MSCs, consistent with the improvement in renal function and morphology.^31^ Melatonin, which was used as a dietary complement in humans, also presented potential to promote MSCs survival. In a rat I/R renal failure model, Mias et  al showed that melatonin pretreatment strongly increased the survival of MSCs after intraparenchymal injection. Surviving MSCs further induced overstimulation of angiogenesis, proliferation of renal cells and accelerated recovery of renal function.^27^ Similarly, preconditioning with muscone, the main active ingredient of musk, also enhanced the proliferative ability of bone marrow‐derived mesenchymal stem cells (BMSCs) to some degree in rats with gentamicin‐induced AKI.^32^

Lastly, many studies have confirmed preconditioning with insulin‐like growth factor‐1 (IGF‐1) may enhance MSC proliferation with lower apoptosis in many other organ failure models.^33^, ^34^ In AKI, Xinaris et  al found that the number of IGF‐1‐treated MSCs was increased in the injured kidney at day 1 and remained higher at day 4, partly due to the mechanism that preconditioned cells were more resistant to the oxidative damage induced by H_2_O_2 _in vitro.^35^

### 2.2 Improvement of culture condition

During ex vivo expansion, a series of cell‐surface molecules might become barely detectable, causing dysfunction for cell‐cell adhesion.^36^, ^37^ Three‐dimensional (3D) spheroid cultures were reported to promote the expression of surface molecules responsible for cell adhesion and survival.^38^ Using 3D spheroid culture, Xu et  al found that 3D spheroids of MSCs produced higher levels of extracellular matrix (ECM) and had significantly higher expression of the ROS‐scavenging protein Bcl‐2 and prosurvival protein phosphorylated AKT when stimulated by an oxidative condition of H_2_O_2_. After injection into rat models of I/R‐induced AKI, these cells showed enhanced survival rate confirmed by DiI staining, as well as enhanced therapeutic effects of MSCs for AKI.^39^

### 2.3 Thermosensitive hydrogel

After transplantation, MSCs face a harsh environment. Anoikis is very common due to the loss of anchorage‐dependent attachment to the ECM.^40^, ^41^ Approaches were then explored for mimicking a cellular microenvironment more consistent with that found in vivo.

Thermosensitive hydrogel could be an excellent method for improving the microenvironment as well as enhancing the survival rate of transplanted cells.^42^, ^43^ Gao et  al used chitosan chloride hydrogel as a cell carrier for MSC delivery into rat models of I/R‐induced AKI; the authors observed that the hydrogel scaffold could improve the retention and survival of grafted MSCs.^44^ Similarly, in 2016, Feng et  al synthesized an IGF‐1C domain modified chitosan hydrogel. This hydrogel protected cells from H_2_O_2_ treatment and decreased expression of apoptosis‐related genes.^45^

### 2.4 Genetic modification

As discussed above, the imbalance between ROS and antioxidants in the AKI microenvironment was regarded as the main reason for poor cell survival rate. Genetic modification to make MSCs overexpress cytokine genes or antiapoptotic genes significantly improved their survival rate in injured tissues.^46^, ^47^

Heme oxygenase‐1 (HO‐1), a stress‐inducible enzyme that can catalyze the pro‐oxidant heme into biliverdin, CO and free‐iron, exerted powerful antioxidant effects.^48^, ^49^, ^50^ By a gene transfection method, Liu et  al constructed HO‐1 overexpression BMSCs (HO‐1‐BMSCs). While using I/R‐induced AKI kidney homogenate supernatant mimicking the AKI microenvironment, the authors found that HO‐1‐BMSCs showed an improved survival rate. Part of the protective mechanism was due to the antioxidant, anti‐apoptosis and anti‐inflammatory effects of HO‐1 overexpression.^51^ Recently, these authors published a new paper, which documented that modification with HO‐1 significantly attenuated cell‐cycle arrest, activated PI3K/Akt and MEK/ERK pathways and enhanced the survival of MSCs, all of which helped to improve the therapeutic effect of BMSCs on I/R‐induced AKI.^52^

Nrf2 is a transcription factor that activates multiple antioxidant and detoxification enzymes.^53^, ^54^ Mohammadzadeh‐Vardin et  al transiently overexpressed Nrf2 in rat MSCs. The recombined MSCs were more resistant to cisplatin both in vitro and in vivo.^55^

Tissue kallikrein (TK) had pleiotropic effects in protection against oxidative organ damage.^56^, ^57^ Hagiwara et  al demonstrated in their article that combined MSCs and TK gene significantly improved stem cell survival rates during oxidative stress and provided advanced cell viability together with cultured proximal tubular cells.^58^

## 3 STRATEGIES TO ENHANCE MSCS PARACRINE ABILITY

Secretion of paracrine mediators is regarded as the main regenerative mechanism of MSCs in injured tissues. MSCs can secret a variety of cytokines, growth factors and proteins, exerting a wide range of antiapoptotic,^59^, ^60^ immunomodulatory,^61^, ^62^ antioxidative^63^, ^64^ and pro‐angiogenic activities.^65^, ^66^ Similar to the preconditioning strategies mentioned above, some methods have also been explored to enhance MSC paracrine ability after transplantation (Table 2).

**Table 2:** Strategies to enhance MSCs’ paracrine ability in AKI

References	Year	Animal	AKI model	MSCs source	Preconditioning strategy	Outcomes
Zhang et al^67^	2014	Rats	I/R	AMSCs	Hypoxia	↑bFGF and VEGF
Overath et al^68^	2016	Mice	Cisplatin	AMSCs	Hypoxia	↑bFGF, MMP12 and VEGF
Tian et al^29^	2012	Mice	I/R	NM	Incubation with cytokines or chemical compounds	↑HGF and IGF‐1
Masoud et al^30^	2012	Rats	I/R	BMSCs	Incubation with cytokines or chemical compounds	↑IGF‐1 and VEGF
Cai et al^31^	2014	Rats	I/R	BMSCs	Incubation with cytokines or chemical compounds	↑IGF‐1, b‐FGF and HGF
Mias et al^27^	2008	Rats	I/R	BMSCs	Incubation with cytokines or chemical compounds	↑bFGF and HGF
Liu et al^32^	2014	Rats	Gentamicin	BMSCs	Incubation with cytokines or chemical compounds	↑BMP‐7
Xinaris et al^35^	2013	Mice	Cisplatin	BMSCs	Incubation with cytokines or chemical compounds	↑IGF‐1
Bai et al^74^	2017	Mice	I/R	BMSCs	Incubation with cytokines or chemical compounds	↑PGE2
Xu^39^	2016	Rats	I/R	AMSCs	Improvement of culture condition	↑VEGF, bFGF, EGF, HGF, IGF and TSG‐6
Katsuno et al^75^	2013	Rats	Folic acid	AMSCs	Improvement of culture condition	↑HGF and VEGF
Feng et al^45^	2016	Mice	I/R	AMSCs	Thermosensitive hydrogel	↑IGF‐1, HGF and EGF
Liu et al^76^	2013	Mice	I/R	BMSCs	Genetic modification	↑BMP‐7, HGF, and IL‐10
Roudkenar et al^79^	2018	Rats	Cisplatin	BMSCs	Genetic modification	↑HGF, IGF‐1, FGF and VEGF
Hagiwara et al^58^	2008	Rats	I/R	BMSCs	Genetic modification	↑VEGF and recombinant human kallikrein

AKI: acute kidney injury; I/R: ischemia/reperfusion; NM: not mentioned; BMSCs: bone marrow‐derived mesenchymal stem cells; AMSCs: adipose‐derived mesenchymal stem cells; NM: not mentioned; bFGF: basic fibroblast growth factor; VEGF: vascular endothelial growth factor; MMP12: matrix metalloproteinase 12; HGF: hepatocyte growth factor; IGF‐1: insulin‐like growth factor‐1; BMP‐7: bone morphogenetic protein‐7; PGE2: prostaglandin E2; EGF: epidermal growth factor; TSG‐6: tumour necrosis factor‐alpha stimulated gene/protein 6.

### 3.1 Hypoxia

Different from the atmospheric oxygen tension (21%) in a standard cell culture environment, a hypoxic environment is experienced by transplanted MSCs under ischemic conditions. Culturing in oxygen tension that more closely resembles the situation in vivo has been confirmed to help considerably with many aspects, including cell survival, proliferation, differentiation and, most importantly, paracrine ability.

Zhang et  al cultured MSCs in 1% O_2_ hypoxic conditions for 24 hours. The authors found that basic fibroblast growth factor (bFGF) and vascular endothelial growth factor (VEGF) levels were significantly higher in hypoxia‐preconditioned MSCs. These elevated angiogenic factors presented antioxidative, antiapoptotic and angiogenic capacities on I/R‐induced AKI renal cells.^67^ Overath et  al further investigated the paracrine effects of MSCs preincubated in a 0.5% O_2_ hypoxic environment. After analysis using a protein array, they reported expression changes in 64 proteins, including bFGF, VEGF and matrix metalloproteinase 12 (MMP12). An in vivo study also showed that MSC‐conditioned medium significantly ameliorated serum creatinine and the levels of inflammatory cytokines in a mouse model of cisplatin‐induced AKI.^68^

### 3.2 Incubation with cytokines or chemical compounds

In addition to the protective effect mentioned above, incubation with cytokines or chemical compounds also enhanced the MSC paracrine effect. Treatment of MSCs with 14S,21R‐diHDHA promoted secretion of hepatocyte growth factor (HGF) and IGF‐1.^29^ The expression of IGF‐1 and VEGF genes also revealed many fold increases after preconditioning with SNAP.^30^ For drugs or health care products, Cai et  al demonstrated that MSCs pretreated with atorvastatin expressed higher levels of IGF‐1, bFGF and HGF,^31^ similar to the results obtained by Mias et  al, who incubated MSCs with melatonin.^27^ Liu et  al used muscone to precondition MSCs, and higher levels of bone morphogenetic protein‐7 (BMP‐7) were observed both by real‐time qPCR and ELISA.^32^ Interestingly, Xinaris et  al pretreated MSCs with IGF‐1. After full washout, they still observed a 10‐fold increase of IGF‐1 in the preconditioning group.^35^ MSCs could possibly amplify their regenerative effects through autocrine activity.

Prostaglandin E2 (PGE2) was thought to be important for MSC‐mediated immune modulation.^69^, ^70^ PGE2 secretion could increase the proportion of Tregs, which could inhibit effector T cells both in vitro and in vivo, presenting an anti‐inflammatory effect.^71^, ^72^, ^73^ Bai et  al pretreated MSCs with a cytokine, IL‐17A, and then transplanted them into rat models of I/R‐induced AKI. Significantly lower acute tubular necrosis scores, serum creatinine and BUN were observed in mice that received pretreated MSCs therapy. They further demonstrated that the better therapeutic efficacy was due to the increase of Treg percentages through the COX‐2/PGE2 pathway.^74^

### 3.3 Improvement of culture condition

Improved culture conditions were also beneficial for MSCs’ paracrine ability. 3D spheroid culture increased the secretion of VEGF, bFGF, epidermal growth factor (EGF), HGF, IGF and tumour necrosis factor‐alpha stimulated gene/protein 6 (TSG‐6).^39^ In 2013, Katsuno et  al cultured MSCs in a low serum culture medium containing (2% fetal bovine serum) (lMSCs). They observed that lMSCs secreted higher levels of HGF and VEGF compared to the MSCs cultured in high serum (hMSCs). After transplantation into rat models of folic acid‐induced AKI, lMSCs significantly attenuated acute renal damage and showed less interstitial fibrosis change on day 14.^75^

### 3.4 Thermosensitive hydrogel

Thermosensitive hydrogels not only helped MSC survival but also improved their paracrine ability. Feng et  al demonstrated in their article that using an IGF‐1C domain modified chitosan hydrogel could help MSCs up‐regulate the expression of IGF‐1, HGF and EGF.^45^

### 3.5 Genetic modification

Compared with other preconditioning strategies, genetic modification is a more accurate way to enhance MSC paracrine ability. Overexpression of chemokine (C‐X‐C motif) receptor 4 (CXCR4) in BMSCs, using lentivirus vector, resulted in higher levels of BMP‐7, HGF and IL‐10. Significantly improved renal function, reduced ATN scoring, more PCNA tubular cells and fewer TUNEL tubular cells were also observed using in vivo studies.^76^ Lipocalin 2 (Lcn2) was thought to be a cytoprotective factor against AKI due to its important role in regeneration and proliferation of tubular epithelial cells.^77^, ^78^ Roudkenar et  al genetically manipulated MSCs to upregulate Lcn2. Their results revealed that upregulation of Lcn2 not only significantly stimulated the secretion of HGF, IGF‐1, FGF and VEGF in MSCs but also ameliorated renal dysfunction caused by cisplatin‐induced AKI.^79^ Lastly, the article by Hagiwara et  al suggested kallikrein‐modified MSCs were also able to secrete recombinant human kallikrein with elevated VEGF levels in culture medium.^58^

## 4 NEED FOR A STANDARD MSCS REGIMEN FOR AKI THERAPY

Despite the multiple lines of evidence, there is still no standard MSCs regimen regarding the best tissue source, cell type, delivery route, dosage and timing for AKI therapy, due to the high heterogeneity of MSCs and the pathophysiological complexity of AKI. A standard regimen using MSCs for AKI therapy needs to be established for the successful translation of MSC applications from preclinical research to clinical trials.

First, which type of MSC is the best one for AKI patients? Thus far, BMSCs are still the most widely used MSCs in various animal AKI models and the only one that has proven effectiveness in clinical studies. Most included studies in our review are also relevant with BMSCs. However, BMSCs have their own limitations, such as a relatively invasive collective method and restricted quantities. Instead, the characteristics of noninvasive collection together with the larger quantities for foetal membrane MSCs (FM‐MSCs) and the greater survival rates for cord blood‐MSCs (CB‐MSCs) present their advantage for AKI treatment.^80^, ^81^ Recently, accumulating evidence in the area of anthracycline‐induced cardiomyopathy and limb ischemia injury showed that induced pluripotent stem cell derived MSCs (iPSC‐MSCs) exhibited better therapeutic effects over BMSCs. iPSC‐MSCs not only overexpressed macrophage migration inhibitory factor (MIF) and growth differentiation factor‐15 (GDF‐15) but also presented superior efficiency of mitochondrial transfer than BMSCs, which protected cardiomyocytes against anthracycline‐induced damage.^82^, ^83^ In a mouse model of hind limb ischemia, it was demonstrated that transplanted iPSC‐MSCs revealed markedly higher survival rates and less inflammatory cell infiltration, which eventually resulted in better limb function than BMSCs. The authors then documented that iPSC‐MSCs poorly expressed HLA‐II after either proinflammatory IFN‐γ stimulation or transplantation, confirming the stronger immune privilege of iPSC‐MSCs.^84^ iPSC‐MSCs are single colony cell line MSCs derived from iPSCs under certain conditions. The self‐renewal ability of iPSCs makes them an unlimited and noninvasive source of MSCs.^85^ The application of iPSC‐MSCs can also solve some clinical bottleneck problems, such as the heterogeneity of acquired MSCs and the aging‐related disorders of donor cells. However, the potential risk of tumour formation due to the use of reprogramming factors to induce pluripotency remains a clinical concern, although some methods without viral vectors have already been developed. The application of iPSC‐MSCs in I/R‐induced AKI was also reported in 2017,^86^ but we still need more research to answer the question of whether iPSC‐MSCs or another type of MSCs is the best one for AKI patients.

Second, although preconditioning strategies have been regarded as powerful approaches for improving the survival rate and paracrine ability of MSCs, the specific signalling pathway is still not very clear. Among these diverse pathways, nuclear factor‐kappa B (NF‐κB) is a transcription factor that widely participates in cellular processes through modulating gene expression. NF‐κB in MSCs can be activated by a range of stimulation, including cytokines and stress stimuli such as TNF‐α, LPS and hypoxia, leading to the over production of multiple growth factors, including VEGF, HGF, FGF2 and IGF‐1.^87^, ^88^ Bai et  al demonstrated that preconditioning with TNF‐α in MSCs could promote their survival and migratory abilities together with an increase in the phosphorylation of NF‐κB‐p65. They further showed that these effects could be partially abolished by IKK XII (NF‐κB inhibitor), indicating the role of NF‐κB in regulating cell viability and migration.^89^ These studies confirmed that the activation of NF‐κB might be involved in the cytoprotective, migratory and paracrine processes of MSCs. In contrast, decreased NF‐κB activity was observed in Rap1‐/‐BMSCs, which displayed more resistance to apoptosis and presented better cardioprotective effects in myocardial infarction mice than wild‐type BMSCs.^90^ Similarly, the results from mice fed a high fat diet (HFD) identified HFD‐induced activation of NF‐κB in MSCs, contributing to the reduced expression of VEGFA and bFGF.^91^ In conclusion, the exact role NF‐κB plays in MSCs is still debated. Although most studies support the activation of NF‐κB to maximize the therapeutic effects of MSCs, more research associated with the relationship between NF‐κB and MSCs function may help to solve this problem.

Third, whether extracellular vesicles (EVs) derived from MSCs are able to substitute for MSCs still needs further confirmation. EVs are a population of heterogeneous vesicles that can be released to the extracellular space by MSCs. According to the size, EVs can be divided into exosomes (30‐100 nm diameter) and microvesicles (MVs) (100 nm‐1 μm diameter). The first article that reported the beneficial effect of EVs in the area of AKI was by Bruno et  al in 2009. In a glycerol‐induced AKI model, they found that EVs significantly accelerated the morphologic and functional recovery of injured kidneys.^92^ Subsequently, a series of studies confirmed the therapeutic effect of EVs for AKI. Although the specific mechanism is still not entirely clear, it is said that EVs are enriched in proteins, lipids, mRNAs, miRNA and organelles, which can modulate selective cellular pathways in injured cells through cell‐to‐cell communication, presenting trophic and reparative effects.^93^ Because they are cell‐free, treatment with EVs is thought to be safer than direct delivery of MSCs in terms of tumourigenicity. The problems of storage, sterilization and potency assays are also much easier for EVs. However, it appears that not every subpopulation of EVs may have the same effects. Burger et  al demonstrated that in a hypoxia/reoxygenation‐induced endothelial cell injury model, conditioned medium or exosomes derived from endothelial colony‐forming cells (ECFCs) significantly relieved apoptosis, while MVs were ineffective.^94^

The fourth question is the route of MSCs delivery. Because the therapeutic effects of MSCs for AKI mainly rely on their paracrine/autocrine ability, it is very important to deliver MSCs to injured tissues. Intravenous injection, intra‐arterial injection, intraperitoneal injection and intrarenal injection are the four main delivery methods in animal models. In 2013, a meta‐analysis demonstrated that intra‐arterial injection of MSCs induced greater decrease of elevated Scr compared with intrarenal injection and intravenous injection.^95^ However, the studies included in this meta‐analysis are a combination of chronic kidney disease and AKI, which may cause confounding bias. Therefore, in 2016 Zhang et  al conducted a meta‐analysis that analyzed the effects of intravenous injection of MSCs‐derived EVs with other routes of delivery in AKI models.^96^ After multivariable meta‐regression analysis, they concluded no difference in SCr reduction was found between different delivery routes. These confusing results may come from the different types of cells used in these studies. Intravenously delivered MSCs might be retained in the lung capillaries, which reduced their therapeutic effects, while the smaller size of EVs made them easily pass through lung capillaries and migrate towards injured tissue, presenting similar therapeutic effects as intra‐arterial injection.^92^, ^97^ Due to the limitations of these meta‐analyses, it is still difficult to say which delivery route is the best for AKI management. We need more studies to answer this question.

Last but not the least, the issue of humoural or cellular responses should still be considered for the clinical application of MSCs. Until now, only one study compared the safety and efficacy of allogeneic with autologous MSCs in AKI models. Different doses of allogeneic and autologous MSCs were infused in rats with I/R‐induced AKI. Allogeneic and autologous MSCs both presented immediate renoprotective effects. After a 3‐month follow‐up period, renal function was preserved and no significant MSC‐induced side effects occurred in both groups. In addition to the safety and efficacy of allogeneic MSCs, a panel of fibrosis‐related genes were decreased in the allogeneic group, demonstrating the potential better therapeutic effects of allogeneic MSCs.^98^ Similar results were obtained from POSEIDON‐DCM trial, which demonstrated the safety and supported the greater benefit of allogeneic than autologous MSCs for nonischemic dilated cardiomyopathy patients.^99^ Allogeneic MSCs can be advantageous to autologous ones for a number of reasons, such as low expenses, high quality and easy processes during the harvesting period.^100^ The main concern of allogeneic MSC application arises from their potential antigenicity. Due to the absence of major histocompatibility class II antigens (MHC II) on the cell surface, MSCs can be regarded as an immunoprivileged property in vivo. What is more, some researchers even speculate that the rejection of MSCs is a way to activate a regulatory immune response and exert therapeutic benefits.^101^ Conversely, in an equine model, compared with autologous MSCs, repeated intra‐articular injection of allogeneic MSCs resulted in an adverse clinical response, suggesting adaptive immunity may occur in response to repeated exposure.^102^ Factors such as dosage, timing, route and tissue source may have an impact on this issue and induce discordances in both in vivo and in vitro settings.

## 5 CONCLUSION AND FUTURE PERSPECTIVE

In conclusion, how much of a role MSCs exert clinically in AKI largely depends on their survival, paracrine and migration ability. Without a doubt, various preconditioning strategies improve MSCs survival and paracrine ability, exaggerating their beneficial effects. Due to the huge heterogeneity in MSCs therapeutic regimens, it is difficult to demonstrate which preconditioning strategy is the best one. Different donors, sources, route timing and doses of transplanted MSCs may need different preconditioning strategies. The advantages and disadvantages of different preconditioning strategies mentioned in this article are listed in Table 3.^19^, ^103^, ^104^, ^105^, ^106^, ^107^, ^108^ A better understanding of the molecular and cellular mechanisms underlying the preconditioning strategy may help considerably to expand its application in AKI and avoid potential side effect. A preconditioning strategy that can not only enhance the survival, paracrine and migration ability of MSCs but also have no potential adverse effect is the most suitable preconditioning strategy for AKI therapy. We look forward to an optimistic future of MSCs therapy in AKI and call for more research in this area.

**Table 3:** Advantages and drawbacks of different preconditioning strategies mentioned in this article

References	Preconditioning strategy	Advantages	Drawbacks
Silva et al^19^	Incubation with cytokines or chemical compounds	Simple and fast	Risk of changes in gene expression
Hu and Li^103^	Improvement of culture condition	Simple and fast	Optimization problem
Li et al^104^	Thermosensitive hydrogel	Biocompatible and targeted	Difficult and expensive; Hydrogel solidification process may do harm to MSCs
Hu and Park^105^, ^106^	Genetic modification	Accurate	Complex and expensive; Vector toxicity; Low transfection efficiencies; Potential tumourigenicity
Ruud and Tsai^107^, ^108^	Hypoxia	Simple and safe	Discrepancies on a standardized protocol; Technical limitations for measuring the accurate oxygen tension experienced by the cells

## CONFLICT OF INTEREST

The authors confirm that there are no conflicts of interest.
